# TSP, a virulent Podovirus, can control the growth of *Staphylococcus aureus* for 12 h

**DOI:** 10.1038/s41598-022-13584-5

**Published:** 2022-06-15

**Authors:** Rabia Tabassum, Abdul Basit, Iqbal Ahmed Alvi, Muhammad Asif, Shafiq ur Rehman

**Affiliations:** 1 Institute of Microbiology and Molecular Genetics, University of the Punjab, Lahore, Pakistan; 2 Department of Microbiology, Hazara University, Mansehra, KPK Pakistan; 3 Department of Pathology, King Edward Medical University, Lahore, Pakistan

**Keywords:** Microbiology, Bacteriophages

## Abstract

Methicillin-resistant *Staphylococcus aureus* (MRSA) is a prevailing nosocomial pathogen that is increasingly isolated in community settings. It shows resistance against all beta-lactam drugs and has acquired mechanisms to resist other groups of antibiotics. To tackle this emerging issue of MRSA, there is an urgent need for antibiotic alternatives, and utilizing lytic bacteriophages is one of the most promising therapeutic approaches. In the present study, a lytic bacteriophage TSP was isolated from hospital wastewater against MRSA. The phage efficiently inhibited bacterial growth for up to 12 h at MOI of 1 and 10. TSP phage showed activity against various isolates of MRSA and MSSA, isolated from different clinical samples, with variable antibiotic susceptibility patterns. The bacteriophage TSP showed stability at varying temperatures (25 °C, 37 °C) and pH values (5–9), while its maximum storage stability was observed at 4 °C. It had a short latent period (20 min) and burst size of 103 ± 5pfu/infected cells. TSP genome sequence and restriction analysis revealed that its genome has a linear confirmation and length of 17,987 bp with an average GC content of 29.7%. According to comparative genomic analysis and phylogenetic tree,TSP phage can be considered a member of genus “P68viruses”. The strong lytic activity and short latent period in addition to its lytic nature makes it a good candidate for phage therapy against MRSA infections, if it proves to be effective in in-vivo studies.

## Introduction

Antibiotic resistance is one of the major issues that limits effective treatment against bacterial infections^1^. *Staphylococcus aureus (S. aureus*) is one of the most important gram-positive antimicrobial-resistant pathogens that causes a number of clinical infections, including skin and soft tissues, infective endocarditis, bacteremia, and device-related and respiratory infections, both in nosocomial and community settings^2^. Methicillin resistant *Staphylococcus aureus* (MRSA)exhibits resistance to all beta-lactam antibiotics by virtue of mutated penicillin binding protein, which does not permit binding of the drug with bacterial cells^3^. This organism is also resistant to aminoglycosides, macrolides, fluoroquinolones, chloramphenicol, and tetracycline^4^. MRSA infections account for more than 50% of infections in hospital settings^5^.Treatment failure due to antimicrobial resistance can lead to the development of chronic infections, which not only leads to increased morbidity and mortality but also prolongs hospital stays and higher health care costs compared with methicillin-*sensitive Staphylococcus aureus* (MSSA) infections^6^. MRSA has become a challenging pathogen because it poses a serious threat for hospitals and communities. Therefore, it is of great concern to develop new strategies that can supplement or replace the utility of existing antibiotics for the treatment of MRSA infections.

To overcome the problem of antimicrobial resistance, different alternative strategies can be used, including the use of bacteriophages, monoclonal antibodies, probiotics and antimicrobial peptides. Among all alternative approaches, bacteriophage therapy is the best alternative approach that can be used to treat multiple drug-resistant *S. aureus* infections. The properties that make bacteriophage therapy the best replacement option include safety, high specificity, and effective lytic activity against bacterial cells^7^. Compared to the synthesis of new antibiotics, the production of bacteriophages is cheaper and faster, and bacteriophages can easily proliferate at infection sites with limited or no side effects^8^. *S. aureus* phages have efficient antimicrobial activity, as described in various in vitro and in vivo studies^9^. Phage SLPW and CSA13 isolated from chicken and fecal sewage of pig farms showed 90% and 92% lytic spectra, respectively, against methicillin-resistant *S. aureus* strains. In addition, CSA13 successfully removed*S. aureus* biofilm^10^, while SLPW showed the ability to cure MRSA infection in mice^7^. Furthermore, phages S24-1 and S13 were isolated from sewage and showed 100% and 89% lytic spectra against clinical isolates of *S. aureus*^11^, respectively. The current study describes the isolation and detailed characterization of lytic TSP phage against *S. aureus*, including virion architecture, host range, burst size, latency period, thermal and pH stability, and complete genome sequence analysis.

## Material and methods

### Bacterial strains and culturing media

A total of 42 *S. aureus* clinical isolates were isolated from various clinical samples by our lab and characterized for both phenotypic and genotypic characteristics^3^. The strains were presumptively identified as *S. aureus* by colony morphology on tryptic soya agar (TSA). Pure isolates of *S aureus* were further confirmed by Gram staining and conventional biochemical tests (catalase, DNase, coagulase and mannitol fermentation) as described earlier^12^. For phenotypic identification of MRSA, antibiotic susceptibility testing was performed by modified Kirby Bauer’s Disk diffusion method on Muller Hinton agar with commercially available cefoxitin (30ug) disc. The diameter of inhibitionzone was measured and the results were interpreted according to CLSI 20 criteria^13^. Among them 32 isolates were MRSA, while ten isolates were methicillin sensitive *S. aureus *(MSSA). We also obtained ATCC6538 strain of *S. aureus *and used as control. These strains were further tested for antibiotic sensitivity against a broad range of antibiotics according to CLSI 20 criteria.

### Isolation of bacteriophage

Initially, MR10 isolate^3^ of *S. aureus *was selected randomly as a host for the isolation of bacteriophages from hospital wastewater effluent, Lahore, Pakistan, according to a previously reported procedure^14^. The sample was centrifuged (10,000 rpm, 10 min), and the supernatant was filtered (0.45 µm). Subsequently, 25 mL of the filtrate was enriched with an equal amount of 2X tryptone soya broth (TSB) containing 10 mM CaCl_2_ and 2 mL of fresh bacterial culture (4 h old) and incubated overnight at 37 °C with constant shaking (160 rpm). After incubation, 1% chloroform was added, the flask was left unshaken for half an hour at 37 °C and centrifuged (10,000 rpm, 10 min), and the supernatant was filtered (0.22 µm). The filtrate was assessed for lytic activity by spot test and agar overlay method. For the spot test, 100 µl of log phase host bacterium was mixed with 3–4 mL of 0.7% TSB agar and poured on to a TSB agar plate. A 5 µl aliquot of phage lysate was spotted on a bacterial lawn followed by overnight incubation at 37 °C. Based on lawn clearing, spot test results were categorized as (−) no lytic activity, (+) small lytic activity (hazy clear zone), (++) moderate lytic activity (clear zone with faintly hazy background), and (+++) strong lytic activity (complete clear zone). For the agar overlay method, serial dilutions of phage lysate were prepared in TSB broth, and 100 µl of log phase host bacterium was mixed in each dilution followed by incubation at 37 °C for 30 min. Then, the mixture was mixed with 3–4 mL of 0.7% TSB agar and spread on TSB agar plates. After overnight incubation at 37 °C, the presence of plaques was observed. Purification of bacteriophages was achieved by picking and replating the well-isolated plaque five times. Enumeration of bacteriophages present in the lysate (bacteriophage titer) per milliliter was performed by counting the number of plaques produced from serial dilutions and reported as plaque forming units (pfu) per milliliter (pfu/mL)^15^.Purified phage was stored in TSB broth at 4 °C as a stock for further characterization experiments. All experiments were run in triplicates.

### Determination of TSP morphology by TEM

Transmission electron microscopy (TEM) was used to analyze the morphology of isolated phage (TSP phage). The purified and concentrated phage preparation was placed on a carbon-coated copper grid for 15 min and then negatively stained with 2% uranylacetate (pH 4.0). Morphology of the TSP phage was examined using TEM at an acceleration voltage of 80 kV. The guidelines of the International Committee on Taxonomy of Viruses were used for the classification of TSP phages.

### Determination of in vitro bacteriolytic activity of TSP

The bacteriophage activity against different bacterial isolates was determined by standard spot assay as described above. A collection of 32 MRSA strains, 8 MSSA, 4 *Staphylococcus epidermidis* strains and some species of Gram-negative organisms (*Escherichia. coli*, *Klebsiella pneumoniae, Serratia marcescens Pseudomonas aeruginosa, Acinetobacter baumannii* and *Enterobacter cloacae) *were used to assess the host range. The efficiency of plating (EOP) was calculated as the ratio of bacteriophage particles obtained from susceptible bacteria to bacteriophages obtained from the original host bacterium (MR10).The bacteriolytic activity of TSP phage was determined by a previously reported method^14^.Briefly, four flasks containing 50 mL of TSB broth were inoculated with 1 mL of overnight-grown bacterial culture (3 × 10^9^ cfu/mL). TSP phage lysate was added at MOI of 10, 1, and 0.1 to three different flasks. TSP phage lysate stock containing 2.4 × 10^10^ pfu/mL was used. To adjust the MOI-10 and 1, 1250 µl and 125 µl of phage stock were used, which correspond to 3 × 10^10^ and 3 × 10^9^pfu*,* respectively. To adjust the MOI-0.1, 125 µl of a 1:10 dilution of phage stock was added, which corresponded to 3 × 10^8^ pfupfu. The fourth flask with only MR10 processed under similar conditions, was considered as negative control. The absorbance (OD_600_) of control and test cultures was assessed for 24 h with an interval of 2 h.

### Determination of bacteriophage stability

The long-term storage stability of TSP phage was determined by incubating phage lysate (1 mL) at different temperatures (4, 25, − 20 and − 80 °C) for 1 month as described earlier^16^. Glycerol (5%) was added to the phage lysate before preservation at − 20 °C and − 80 °C. The bacteriophage titer was determined before and after storage by double-layer agar technique. Short-term bacteriophage stability at different temperatures (25, 37, 45, 50, and 60 °C) and a wide range of pH values (4, 5, 6, 7, 8, 9, and 10) were assessed by treating the known titer of phages for 1 h at the above temperatures and pH. The pH was neutralized to 7.0 followed by titer determination by the agar overlay method^17^.

### Determination of adsorption assay

To determine the time taken by the TSP phage to adsorb to the host surface, an adsorption assay was performed as described earlier with some modifications^18^. Phage adsorption was assayed at MOI of 0.1. Percentages of unadsorbed phages were determined at every 3-min intervals by taking the ratio of pfu/mL to the initial pfu/mL at 0 min in the supernatant. The bacteriophage adsorption rate constant was determined by mathematical formula; K = (2.3/B × t) × log (Po/P)^19^, where B is bacterial density, t is time in minutes, Po is initial phage concentration and P is titer of free phages at a particular time.

### One-step growth curve

To determine different phases of the bacteriophage lytic cycle and burst size, one-step growth curve analysis was performed according to a protocol described previously with some modifications^15^. Briefly, known titer of phage lysate (2.4 × 10^10^ pfu/mL) was mixed with log phase host bacterium to obtain an MOI of 0.01 followed by incubation for 1 min at 37 °C. Centrifugation was carried out at 8000 × g for 10 min to remove the un-adsorbed phages. The pallet was resuspended in 100 mL of TSB broth and incubated at 37 °C with continuous shaking at 150 rpm. After every 5-min interval, 1 mL of sample was withdrawn for up to 60 min at 10 min of interval and harvested. The supernatant obtained was subjected to plating by the overlay agar technique to enumerate the released phage particles. The bacteriophage latent period was determined by plotting the burst size with post incubation time, and the burst size was determined as the ratio of the mean yield of phages used for bacterial infection to the mean yield of phage particles liberated after infection.

### Analysis of TSP Genome

DNA was extracted from purified phage utilizing optimized protocol^16^ and quantified. Next generation sequencing of phageDNA was performed using Illumina sequencing technique from the University of Minnesota, Genomic Centre (UMGC). Reads were analyzed, trimmed, and assembled by applying CLC genomic workbench 10. Genome annotation was performed by using the online PHASTER (https://phaster.ca/) and RAST server (https://rast.nmpdr.org/). Open reading frames (ORFs) were identified by using GeneMarkS (http://opal.biology.gatech.edu/GeneMark/) and further confirmed through online toolBLASTp (http://www.ncbi.nlm.nih.gov/BLAST).  ARNold was used for the detection of potential rho-independent terminators^20^. tRNA Scan-SE software was applied for the prediction of putative tRNAs^21,22^. The molecular weight of proteins was determined using the ExPASy tool (https://web.expasy.org/compute_pi/). The genomic map was constructed using Snapgene software [http://www.snapgene.com/]. The genome sequence was submitted to GenBank under accession number MW286254. Comparative genomic analysis was performed by comparing the whole genome sequence of bacteriophage TSP with other phages of the *Podoviridae*, *Siphoviridae* and *Myoviridae *families through BLASTn^23^. Alignment of sequences and phylogenetic trees were performed in ClustalW^24^. Phylogenetic tree of TSP was determined through online tool Victor^25^ and MEGA7 software^26^ by using whole genome sequences and putative genes that encode major capsids and DNA polymerase.

### Structural analysis of endolysin (ORF7)

To determine the structural variation of TSP endolysin from closely related phages SLPW^7^ and vB_SauP-436A^27^, three-dimensional structures of TSP, SLPW and vB_SauP-436A endolysin were built through the robetta server (available at https://robetta.bakerlab.org/submit.php). The 3D models obtained were further repaired through FoldX to remove side chain clashes. Variations in secondary structural composition were further determined through 2Struc software (available at https://2struc.cryst.bbk.ac.uk/twostruc). The protein–ligand docking of the models was performed through Autodockvina using peptidoglycan ligand obtained from protein complex PDB ID: 4BPA. All the docked structures were analyzed through Pymol.

### Declaration

All experiments were performed in accordance with relevant guidelines and regulations provided by Institute of Microbiology and Molecular Genetics, University of the Punjab, Lahore, Pakistan.

### Compliance with ethical standards

This article does not contain any studies with human participants or live animals performed by any of the authors. However, the clinical samples collected from City lab, Lahore, Pakistan, were already processed for diagnosis and the permission were granted by corresponding lab after getting consent from the patients to proceed the samples from research purpose.

## Results

### S. aureus isolates

Based on the antibiotic sensitivity pattern and genome analysis, the 42 isolates of *S. aureus* from different clinical samples were categorized into MRSA (32isolates) and MSSA (10 isolates)^3^. These isolates when further tested for antibiotic resistance, showed a variable antibiotic resistant pattern against 21 antibiotics (Table S1).

### Morphological characterization of TSP bacteriophage

**Figure 1:**
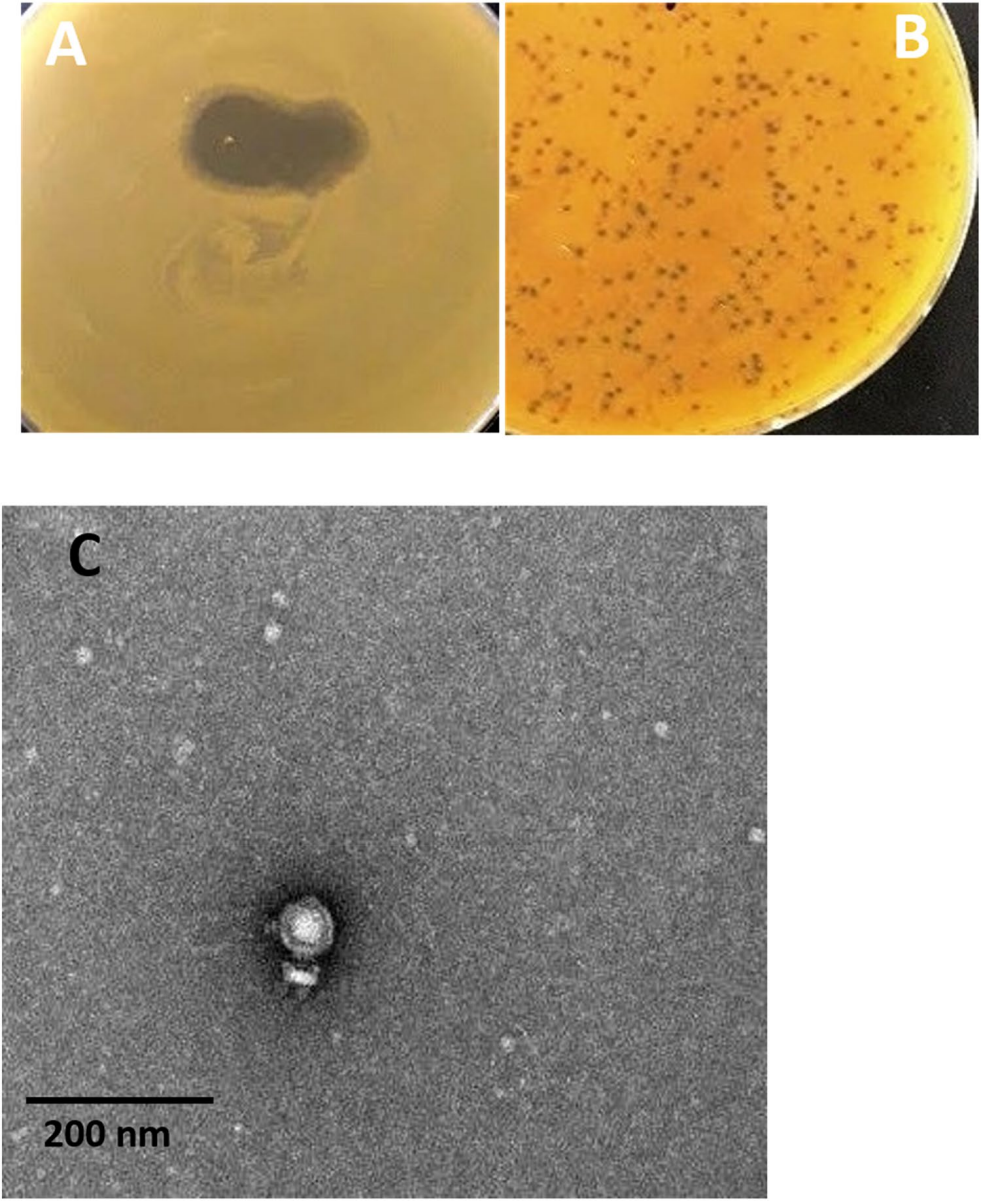
(**A**) The bacteriophage TSP spot on the lawn of MR10. (**B**) Small, circular and lytic plaques of TSP phage obtained by plaque assay (**C**). Transmission electron microscopy of the TSP phage showed its resemblance to the *Podoviridae* family. The scale bar represents 200 nm and image was taken at 25,000X magnification.The uncroped Fig. (**A**) and (**B**) are shown in Fig. S3 in supplementary data.

A lytic phage TSP was successfully isolated from hospital wastewater against *S. aureus*. TSP phage formed tiny clear, round plaques of 1 mm in diameter and a clear spot (Fig. 1A and B). Transmission electron microscopy revealed that TSP phage has a 53 nm icosahedral head with a short non-contractile tail of 22 nm, indicating that TSP phage belongs to the family *Podoviridae* (Fig. 1C).

### Bacteriolytic activity

**Figure 2:**
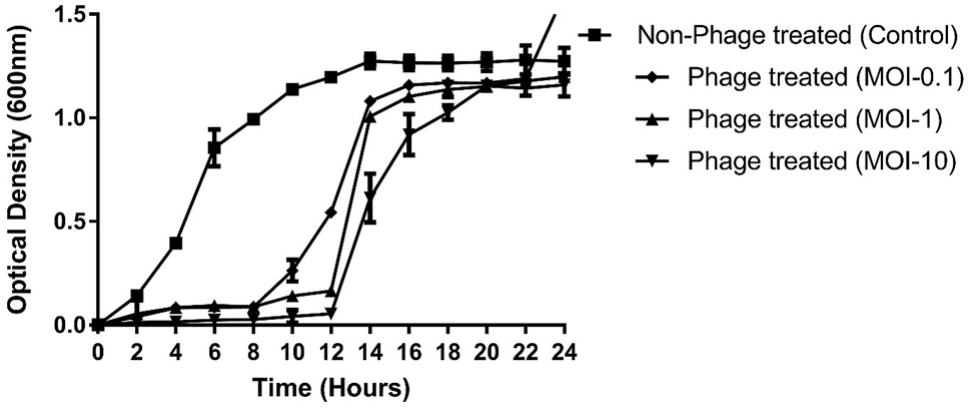
Determination of the invitro bacteriolytic activity of TSP bacteriophage. Phage-treated group: MRSA strain cocultured in logarithmic phase with TSP phage at an MOI of 0.1, MOI-1 and MOI-10. Control group: MRSA culture without phage TSP. The OD of the control and phage-treated groups was measured at 600 nm after an interval of 2 h for up to 24 h. The results are shown as the mean values with standard deviation.

TSP phages showed activity against 24 MRSA and 3 MSSA strains. However, it was unable to infect *S. epidermidis* and other Gram-negative strains used in this study. The efficiency of plating (EOP) of TSP was grouped into four categories^28^: EOP > 0.5 for high production, 0.1 < EOP < 0.5 for medium production, 0.001 < EOP < 0.1 for low production, and EOP < 0.001 for very low production. High EOP production means that infection of the target bacterium produces at least 50% of the pfu in comparison to the primary host.The higher EOP values of the TSP phage against MR5, MR19 and MR26 suggest that these isolates are more susceptible to phage than MR10, while the remaining 15/19 isolates have low plating efficiency compared to the host strain (Table S2). The differences in EOP of TSP against different tested strains might be due to variations in availability of entry receptors and defense mechanisms^29^. TheTSP phage inhibited the host (MR-10) growth for the initial 8 h at an MOI of 0.1 and 12 h at an MOI of 1 and 10, respectively, leading to increased bacterial growth after this time in the phage-treated mixture, but it was still less than the growth in the untreated control (Fig. 2).

### Temperature, pH and storage stability

**Figure 3:**
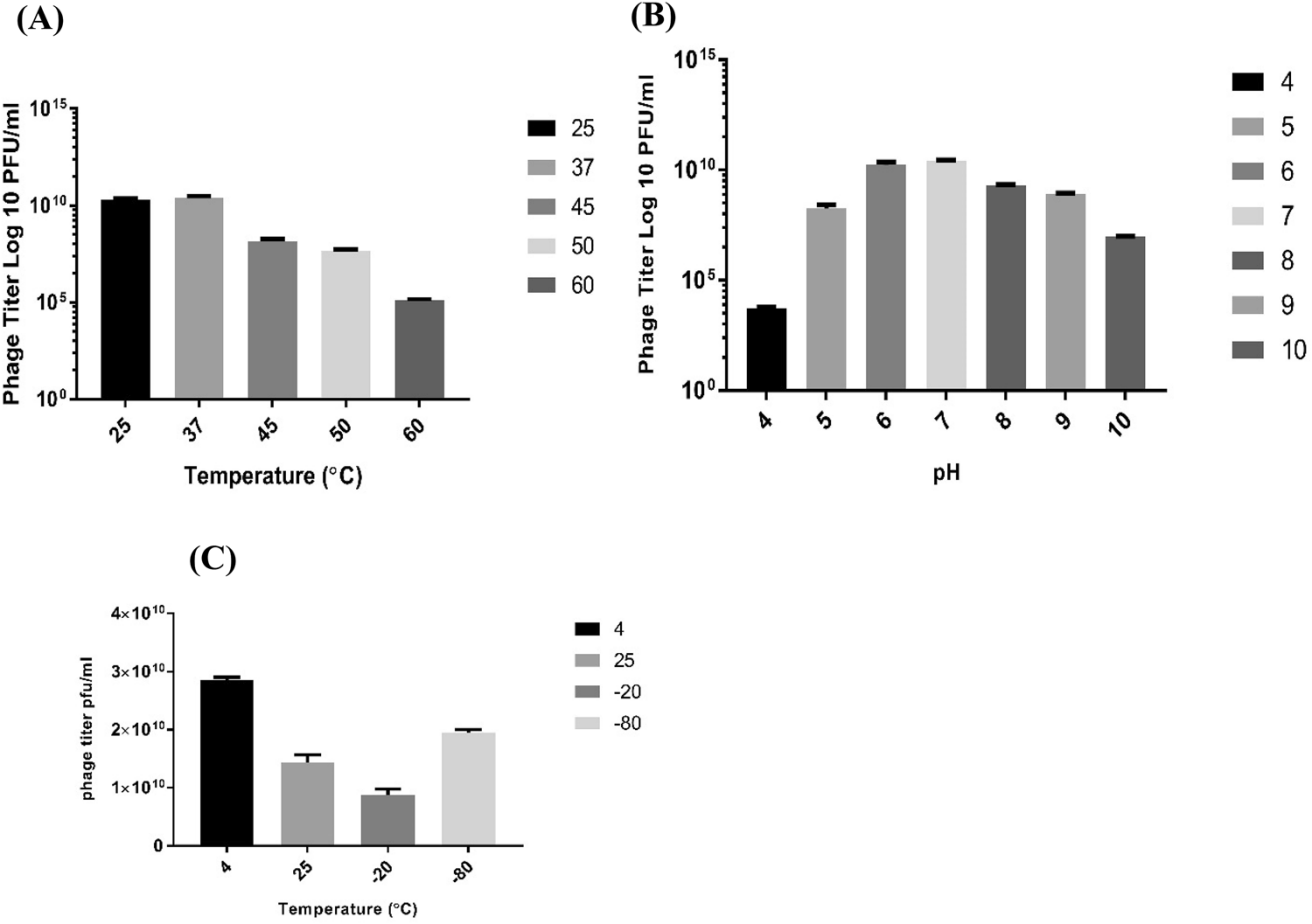
TSP bacteriophage stability assay. (**A**) Effect of different temperatures (25, 37, 45, 50 and 60 °C) on the stability of the TSP phage. (**B**) TSP phage was treated over a wide pH range (4, 5, 6, 7, 8, 9 and 10) for 1 h. (**C**) Storage stability of TSP phage at different temperatures (4, 25,  − 20 and -80 °C) showed maximum survival ability at 4 °C. The experiment was performed three times, and phage titers are expressed as the mean ± standard deviation.

TSP showed short-term stability at temperatures of 25 °C and 37 °C; however, at high temperatures (45 °C, 50 °C and 60 °C), a progressive decrease in phage titer was observed, which destroyed phage activity at temperatures above 60 °C after 1 h incubation (Fig. 3A).The phage remained more active over a wide pH range (5 to 9), but under extreme pH (below 5 and above 10)conditions,a marked decrease in phage titer was observed (Fig. 3B). Long-term storage stability analysisshowed that TSP was more viable at 4°C than at  − 20 °C and  − 80 °C. However, a better survival at − 80 °C (1.95 × 10^10^), while a significant reduction in phage titer was observed at − 20 °C and 25 °C (Fig. 3C).

### Adsorption time and latent period

**Figure 4:**
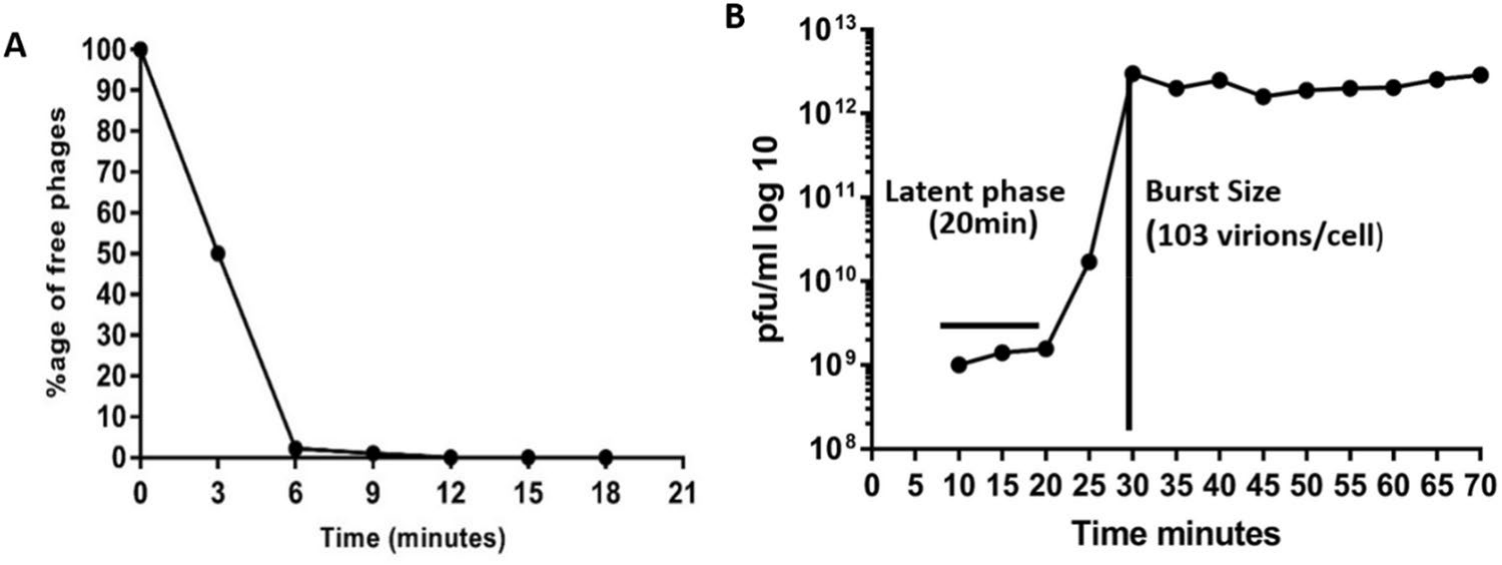
(**A**) TSP phage adsorption kinetics. (**B**) One-step growth curve analysis of bacteriophage TSP infecting MR10 at 37 °C. The results were obtained from three independent experiments.

According to the phage adsorption assay, almost 99% of TSP adsorb to the host cell within initial 6–9 min of infection at 25 °C (Fig. 4A). The adsorption rate constant of phage calculated within the interval of 3–9 min was 4.3 × 10^–12^ pfumL^-1^ min^-1^. One-step growth curve analysis showed a short latency period of 20 min and an average burst size of 103 ± 5virion per infected cell (Fig. 4B).

### TSP has a linear chromosome of 18 kb size

**Figure 5:**
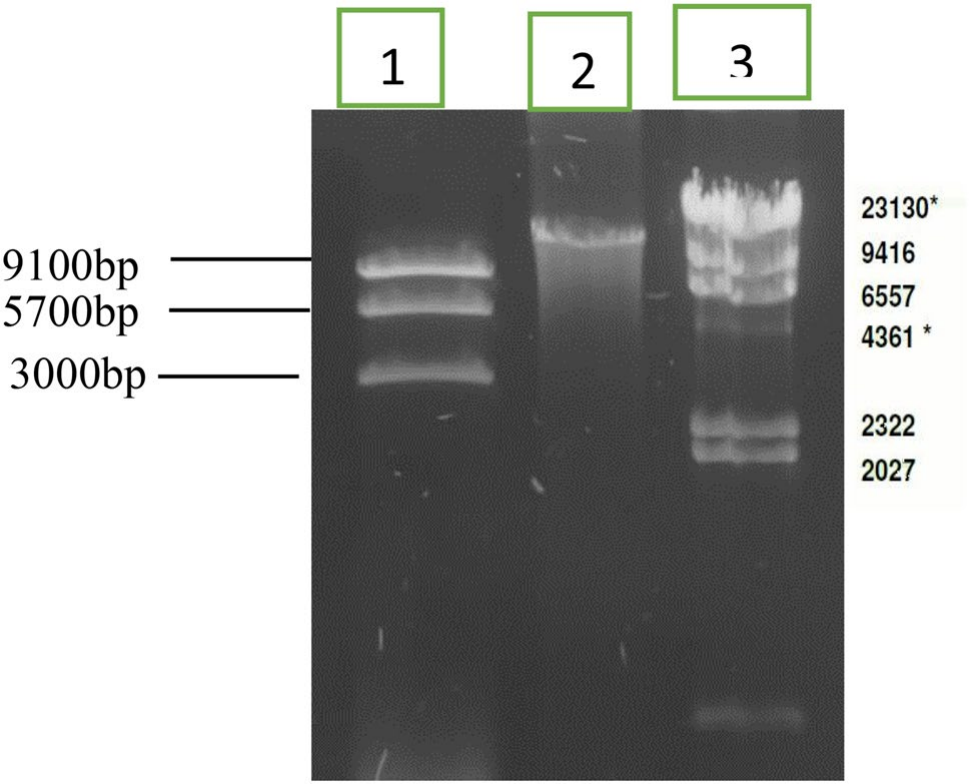
Agarose gel analysis of TSP phage DNA double digested with NcoI and EcoRI. Lane 1: Restricted TSP phage DNA, Lane 2: Unrestricted TSP phage DNA and Lane 3: Lambda phage DNA HindIII digested marker (Cat#302,005 Bioron). An uncroped agrose gel of the above figure isshown in Fig. S4.

To further determine whether the genome of TSB is linear or circular in the packaged state, we identified a customized restriction pattern of the phage genome through Neb cutter and found that restriction through NcoI and EcoRI can produce four fragments of 9.1, 5.7, 3.0 and 0.1 kb sizes, if the genome was linear. Digestion of TSP DNA through NcoI and EcoRI produced a restriction pattern similar to the proposed pattern, suggesting linear configuration of packaged TSP genome (Fig. 5). The theoretical digestion pattern of the TSP genome for linear and circular genomes produced 4 and 3 bands, respectively, through a neb cutter when custom digested through NcoI and EcoRI (Fig. S1).

### Genome sequence analysis demonstrates the lytic nature of TSP phages

**Figure 6:**
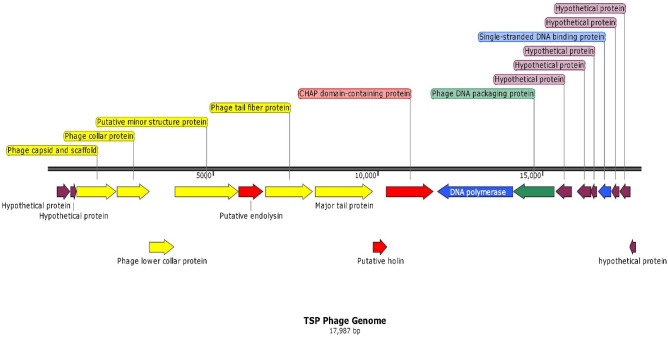
Linear genome map of TSP phage. The direction of ORFs is depicted via arrows. Four functional groups are present in the TSP phage genome, and genes in each functional group are represented by different colors: structural genes (yellow), regulatorygenes (blue), host lysis genes (red), and DNApackaginggenes (green). Hypothetical ORFs are indicated in purple.

Whole genome sequencing and annotation showed thatthe TSP phage consists of double-stranded, linear DNA with a genomic length of 17,987 bp and an average GC content of 29.7%. It contains 20 predicted open reading frames (ORFs)with no tRNA gene. According to BLASTn analysis, the complete TSP phage genome sequence showed 97% identity to *S. aureus* lytic phages SCH1 (Accession No. KY000084.1), SCH11 (Accession No. KY000085.1) and vB SauP-436A1 (Accession No. MN150710.1) with 98% query coverage. The detailed genomic characterization of theTSP phage is given in Supplementary material Table S3. Among all 20 ORFs, 12 had assigned functions, while 8 ORFs were annotated as hypothetical proteins. Annotation and functional analysis of predicted ORFs revealed four functional groups:structural (major capsid and scaffold protein, major and minor tail protein, tail fiber protein, collar proteins, structural protein), host lysis (endolysin, holin and CHAP domain-containing protein), DNA manipulation (single stranded DNA-binding protein, DNA polymerase) and DNA packaging protein. Structural proteins and lysis proteins are present on the plus strand, while DNA metabolism, DNA packaging and maximum hypothetical proteins are present on the negative strand (Fig. 6). TSP phage genome contains 5 potential rho-independent transcription terminators.The virulence finder, an online tool^30^ analysis showed that TSP genome does not contain any virulence gene. According to Pfam and Inter-Pro-Scan analysis, endolysin (ORF7) has two polypeptide domains:a catalytic domain at the N-terminus called cysteine, histidine-dependent amidohydrolases/peptidase (CHAP) (pfam05257) and (IPR007921), and the other is the cell wall binding domain at the C-terminus named SH3_5 (pfam08460) and (IPR003646). TSP phage endolysin is located between structural proteins similar to phage CSA13^10^, and this is a unique characteristic of P68-like viruses.

### Genomic comparison of TSP with *Podoviridae*family phages

**Figure 7:**
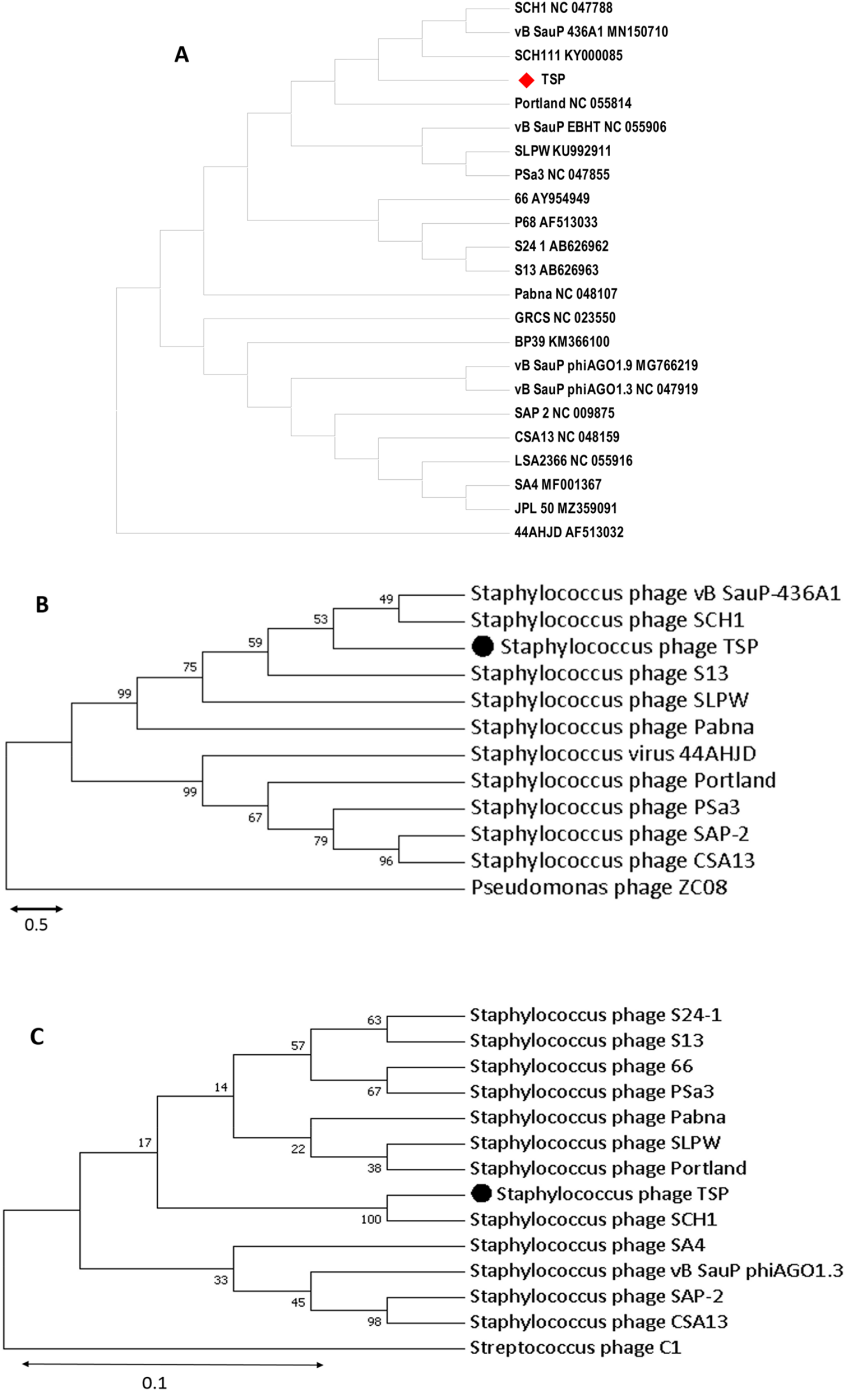
(**A**) Comparative genomic analysis of S*. aureus* phages. Whole genome sequences were used to construct a phylogenetic tree in Victor, an online tool. The red tilted square box highlights the phage TSP. Phylogenetic analysis of TSP based on the amino acid sequences of major capsid (**B**) and DNA polymerase (**C**). Phylogenetic trees for phage proteins were constructed with the alignment tool UPGMA with a bootstrap value of 2000. *Pseudomonas* phage ZC08 and *Streptococcus* phage C1 act as an outgroup. The scale bar represents 0.5 and 0.1 fixed mutations per amino acid position.The dark dot highlights newly isolated phage TSP.

Complete genome sequence of TSP phage was assessed for homology with other *S. aureus* phages (Fig. 7A). Comparative genomic analysis indicated that TSP genome showed the highest homology with the phages of the *Podoviridae* family, while it showed a distant relationship to the members of other families. According to BLASTp search, the major capsid protein of TSP phage showed 99.9% identity to vB SauP-436A1, SCH1 and S13 with 100% query coverage, while the DNA polymerase of the TSP phage showed 98.95% identity to the SCH1 sequence with 100% query coverage (Fig. 7B and C).

### Structural variations of TSP endolysin

**Figure 8:**
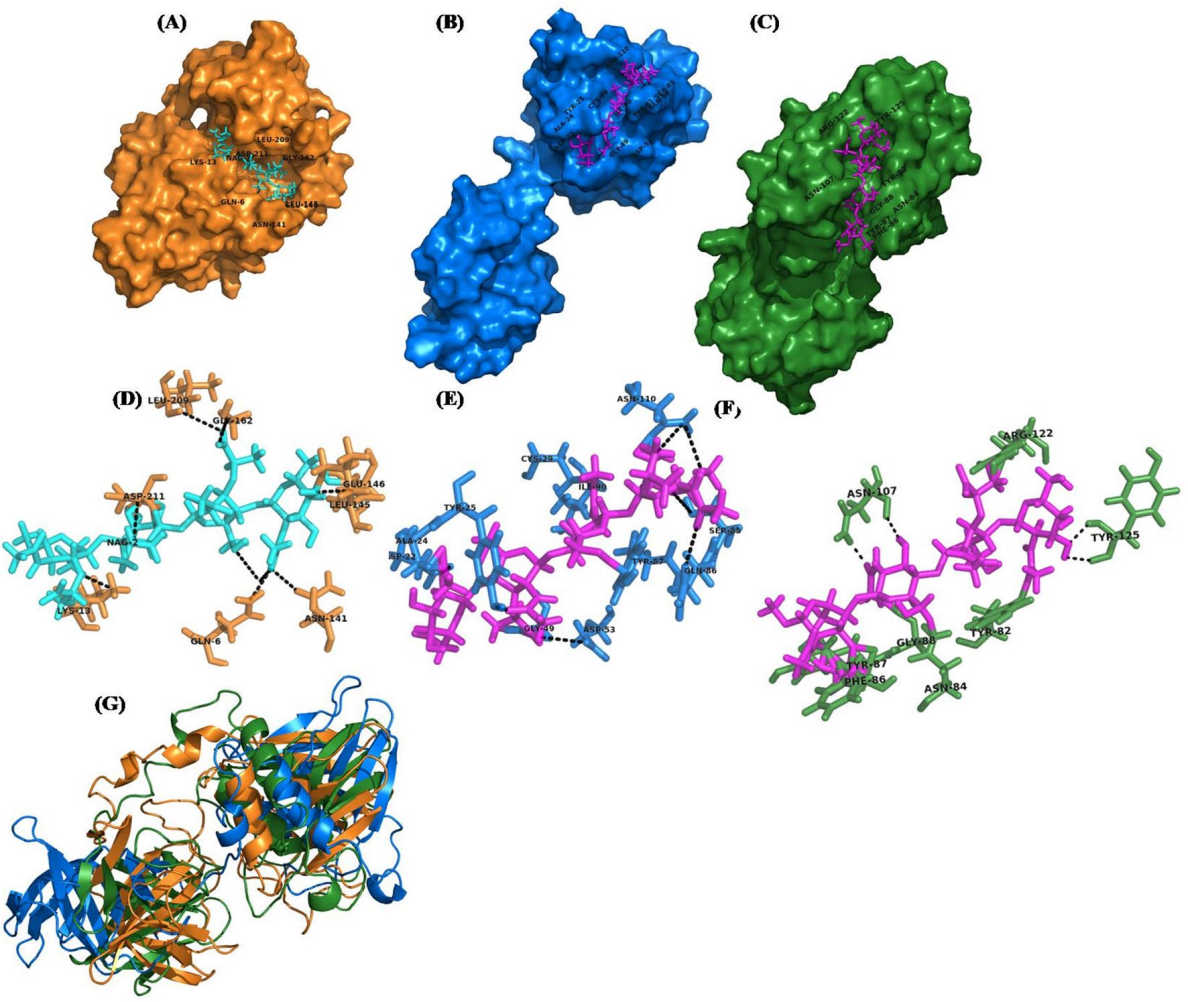
Computational analysis of endolysin structural features from TSP, SLPW and vB_SauP-436A phages. (**A**) Surface model of peptidoglycan ligand (cyan) docked with TSP endolysin (orange), (**B**) SLPW phage endolysin (blue) and (**C**) vB_SauP-436A phage endolysin (green). The surface model of SLPW and vB_SauP-436A phage endolysin showing different conformations, with variation in the position of the docked ligand (pink). (**D**) Most of the residues (orange) involved in binding with ligand (cyan) are located at the C-terminus of TSP endolysin, while (**E**), the SLPW and vB_SauP-436A phage endolysin showed that the N-terminal residues are involved in binding with peptidoglycan ligand (pink) (**G**) The superimposed model of TSP, SLPW and vB_SauP-436A phage endolysin showing different conformations with an RMSD 11–13.5A°.

To determine structural variations of the TSP endolysin and its closely related phages, SLPW and vB_SauP-436A phage, 3D models were built and aligned, which showed 13.5 and 11 A° RMSD, suggesting significant deviation of TSP endolysin structure from that of SLPW and vB_SauP-436A (Fig. 8G). An RMSD > 5A° between two structures represents different conformations^31^. The superimposed models showed that both structures have similar domain compositions; however, there is a difference in linker secondary structure composition as well as conformation of the C-terminal domain (Fig. 8A–F). Secondary structure composition analysis also showed that TSP endolysin consist of 15% helices, while the SLPW and vB_SauP-436A endolysin consist of 11% and 13%, respectively (Table S3). To further investigate the effect of structural composition variation on protein function, protein–ligand docking was performed. Interestingly, the TSP endolysin showed a binding affinity of 6.2 kcal/mol for the peptidoglycan ligand, while SLPW and vB_SauP-436A endolysin showed binding affinities of 7.5 and 7.2 kcal/mol, respectively. Furthermore, the peptidoglycan ligand binds to the N-terminal region of SLPW and vB_SauP-436A endolysin, which is a striking structural feature of modular endolysin targeting Gram-negative pathogens^32^. However, the peptidoglycan ligand bound to the C-terminal residues of TSP endolysin (Fig. 8A–F). The C-terminal region of endolysin plays a vital role in peptidoglycan binding, hence triggering cell lysis of both gram-negative and gram-positive bacteria, even in the absence of holin, suggesting its globular nature^33–35^. Overall, the endolysin of TSP indicates different structural features than that of SLPW and vB_SauP-436A phage.

## Discussion

*S. aureus* is a multidrug-resistant infectious agent responsible for a number of morbidities, such as abscesses, skin infections, endocarditis and toxic shock syndrome^36^. Routine antibiotic therapy has failed to treat infections by MRSA and has become a major challenge in the cure of chronic infections. Currently, the exploration of new strategies to supplement existing antibiotic therapy has become a serious objective of research. In the current era of antibiotic resistance, phage therapy is a suitable alternative solution. Our study aimed to identify virulent bacteriophages against MRSA. A number of studies have been reported on the isolation of bacteriophages from sewage water, which are reservoirs of multidrug-resistant bacteria^37^.

In the current study, TSP exhibited morphology like vB_SauP_436A1, CSA13 and SLPW phages from *Podoviridae* family. Based on morphology and according to the classification of the International Committee on Taxonomy of Viruses, Serogroup C of order Caudovirale can be assigned to this phage^38^. TSP showed lytic spectrum of ~ 75%, suggesting its activity against various MRSA and MSSA isolates from different clinical samples. A strong reduction in bacterial growth was observed until 12 h post incubation by TSP, which is higher than its closely related *S. aureus* phages SLPW lytic period (120 min)^7^, SA97^39^, CSA1 and DW2, which reduced bacterial growth for 3 h^40^. TSP inhibit host growth at lower MOI (1), which is preferred because it might generate lower immune response when applied in living systems^16^.

Long-term stability is also an important parameter for phage preparation used for phage therapy^41^. TSP showed longer survival ability and performance at physiological temperature (37 °C), which favors its therapeutic application against MRSA infections. However, it became inactive at 65 °C, which agreed with decrease in stability of vB_SauS_SA2 and SLPW phages at elevated temperature^42^. TSP exhibits stability over a wide range of pHvalues (5–9) and optimum activity at neutral pH, which agreed with previously reported studies^7,27,43^. Tailed phages mostly maintain virion structure and stability under a wide range of pH values (5–9)^44^. The inactivity of phages below 4 pH indicates that the denaturation of their structural proteins occurs in acidic environments^45^. These characteristics may be helpful inthe administration of phages in different environments as therapeutic agents. We observed that TSP showed optimum storage stability at 4 °C^46^. Our findings confirm that the newly isolated phage TSP is a lytic phage with a short latency period (20 min) and burst size of 103 ± 5virion/infected cells. Its nearest genetic counterpart vB_SauP-436A1 showed a long latent period (50 min) with almost the same burst size of 94 pfu/mL^42^.

Based on genome length, low G + C content and gene organization, the TSP showed similarity with other *S. aureus* lytic phages, VB_SauP-436A1 (97%), SCH1 (97.%), SLPW (93.52%), and bacteriophage 66 (90.67%)^7,27,47,48^.Genes involved in structure, DNA replication, packaging and lysis showed the best match with other *Podoviridae* phages listed in supplementary Table S3^49^. TSP phage also indicated the only characteristic of *S. aureus Podoviridae* phages: DNA packaging and DNA polymerase genes were present on the plus strand, while all structural genes were located on another strand (Table S2), as described earlier^50^. It possess DNA polymerase from the B-type superfamily, which is a unique feature of the Picovirinae subfamily. The classical lysis cassette composed of fused holin-endolysin as single ORF was absent in TSP, similar to other *Podoviruses*^51^, as holin and endolysin are encoded by different ORFs and located at different position in phage genome^52^.

Whole-genome sequences and protein sequences of the major capsid and DNA polymerase were used to infer the evolutionary relationship of TSP phage. Comparative genomic analysis and phylogenetic tree analysis of the TSP phage showed its close relationship to phages SCH1, SCH111 and vB-SauP-436A. TSP phage was taxonomically classified into the Picovirinae subfamily and P68 genus, because it possesses the hallmarks of this subfamily^48^, such as small genome size (16–19 kb), low G + C content (27–29%) and 20–22 predicted number of genes^53^.

## Conclusion

In this study, avirulent bacteriophage TSP was isolated from sewage water against MRSA and characterized. The phage showed bacteriolytic activity against various MRSA and MSSA strains isolated from different clinical samples with variable antibiotic resistance pattern and was capable to inhibit the host growth for 12 h at MOI of 1. The phage persists its activity at room temperature for longer time with a short latency period. The absence of integrase or repressor gene suggest a lytic nature of TSP. Inspite of close relatedness of TSP genome with other *S. aureus* phages, a short latency period, inhibition of host growth for longer period and presence of globular endolysin give it unique characteristics and warrant its application in eradication of *S. aureus* infections, if proven in-vivo.

## Supplementary Information

Supplementary Information.

## Data Availability

The genome sequence has been submitted to the NCBI GenBank database (accession no. MW286254).
