# Supplementary material for: TSP, a virulent Podovirus, can control the growth of Staphylococcus aureus for 12 h
Source: Sci Rep. 2022 Jun 15;12:10008. doi: 10.1038/s41598-022-13584-5 (PMC9200855; doi:10.1038/s41598-022-13584-5)
Supplement: Supplementary file 1 — Supplementary Information. [file 41598_2022_13584_MOESM1_ESM.docx]

**Supplementary Information**

**TSP, a virulent Podovirus can control the growth of *Staphylococcus aureus* till 12 hours**

Rabia Tabassum^1^, Abdul Basit^1^, Iqbal Ahmed Alvi^2^, Muhammad Asif ^1,3^, Shafiq Ur Rehman^1*^

1. Institute of Microbiology and Molecular Genetics, University of the Punjab, Lahore, Pakistan
2. Department of Microbiology, Hazara University Mansehra, KPK Pakistan
3. Department of Pathology, King Edward Medical University, Lahore, Pakistan

**Correspondence:** Dr. ShafiqurRehman, Associate Professor, Department of Microbiology and Molecular Genetics, University of the Punjab, Lahore, Pakistan: email: shafiq.mmg@pu.edu.pk

**Table S1. Antibiotic susceptibility pattern of methicillin resistant *Staphylococcus aureus* (MRSA) isolates from different clinical samples.**

| **Isolates** | **LZD** | **VAN** | **TGC** | **ERY** | **CLI** | **FA** | **SUL** | **OXA** | **CFE** | **PCN** | **FOX** | **CIP** | **IMP** | **SXT** | **NA** | **AMP** | **NOR** | **DO** | **TEC** | **G** | **AMK** |
| --- | --- | --- | --- | --- | --- | --- | --- | --- | --- | --- | --- | --- | --- | --- | --- | --- | --- | --- | --- | --- | --- |
| MR1 | S | S | S | R | R | R | R | R | R | R | R | R | - | - | - | - | - | - | - | S | S |
| MR2 | S | S | S | R | R | R | R | R | R | R | R | R | - | - | - | - | - | - | - | S | S |
| MR3 | S | S | S | R | R | R | R | R | R | R | R | R | - | - | - | - | - | - | - | S | S |
| MR4 | S | S | S | R | R | R | R | R | R | R | R | R | - | - | - | - | - | - | - | S | S |
| MR5 | S | S | S | R | R | R | R | R | R | R | R | R | - | - | - | - | - | - | - | S | S |
| MR6 | S | S | S | R | R | R | R | R | R | R | R | R | - | - | - | - | - | - | - | S | S |
| MR7 | S | S | R | R | R | R | R | R | R | R | R | R | - | - | - | - | - | - | - | S | S |
| MR8 | S | S | S | R | R | R | R | R | R | R | R | R | - | - | - | - | - | - | - | S | S |
| MR9 | S | S | S | R | R | R | R | R | R | R | R | R | - | - | - | - | - | - | - | S | S |
| MR10 | S | S | R | R | R | R | R | R | R | R | R | R | - | - | - | - | - | - | - | S | S |
| MR11 | S | S | R | R | R | S | R | R | R | R | R | R | - | - | - | - | - | - | - | S | S |
| MR12 | S | S | S | R | R | R | R | R | R | R | R | R | - | - | - | - | - | - | - | S | S |
| MR13 | S | S | S | R | R | R | R | R | S | R | R | R | - | - | - | - | - | - | - | S | S |
| MR14 | S | S | S | R | R | R | R | R | R | R | R | R | - | - | - | - | - | - | - | S | S |
| MR15 | S | S | S | R | R | R | R | R | R | R | R | R | - | - | - | - | - | - | - | S | S |
| MR16 | S | S | S | R | S | - | - | - | - | - | R | R | S | R | R | R | - | - | R | S | S |
| MR17 | S | S | S | R | R | - | - | - | - | - | R | R | S | R | R | R | - | - | R | S | S |
| MR18 | S | R | S | R | S | - | - | - | - | - | R | R | S | R | R | R | - | - | R | S | S |
| MR19 | S | S | S | R | S | - | - | - | - | - | R | R | S | R | R | R | - | - | R | S | S |
| MR20 | S | S | S | R | S | - | - | - | - | - | R | R | S | R | - | R | - | - | R | S | S |
| MR21 | S | S | S | R | S | - | - | - | - | - | R | R | S | R | R | R | - | - | R | S | S |
| MR22 | S | S | S | R | S | - | - | - | - | - | R | R | S | R | - | R | - | - | R | S | S |
| MR23 | S | S | S | R | S | - | - | - | - | - | R | R | R | R | S | R | - | - | R | S | S |
| MR24 | S | S | S | R | S | - | - | - | - | - | R | R | S | R | R | R | - | - | R | S | S |
| MR25 | S | S | S | R | S | - | - | - | - | - | R | R | S | R | R | R | - | - | R | S | S |
| MR26 | S | S | R | S | R | R | R | R | R | R | R | R | S | R | R | R | S | S | R | S | S |
| MR27 | S | S | R | R | R | R | R | R | R | R | R | R | S | R | R | R | R | R | R | S | S |
| MR28 | S | S | R | R | R | R | R | R | R | R | R | S | S | R | R | R | R | R | R | R | R |
| MR29 | S | S | R | R | R | R | R | R | R | R | R | R | S | R | R | R | R | R | R | R | R |
| MR30 | R | S | R | R | R | R | R | R | R | R | R | R | S | R | R | R | R | R | R | R | S |
| MR31 | S | S | S | R | R | R | R | R | R | R | R | R | - | - | S | R | R | S | R | S | S |
| MR32 | S | S | S | R | R | R | R | R | R | R | R | R | - | - | S | R | S | R | S | S | S |

**Abbreviations:** LZD= Linezolid,VAN=Vancomycin, TGC= Tigecycline, ERY= Erythromycin, CLI= Clindamycin, FA= Fusidic Acid, SUL= Sulzone, OXA= Oxacillin, CFE= Cefixime, PCN= Penicillin, FOX= Cefoxitin, CIP= Ciprofloxacin, IPM= Imipenem, SXT= Trimethoprim-Sulphamethoxazole, NA= Nalidixic Acid, AMP= Ampicillin, NOR= Norfloxacin, D30= Doxycycline, TEC= Teicoplanin, G= Gentamycin, AMK= Amikacin, R= Resistant, S= Sensitive

**Table S2. Host range and efficiency of plating (EOP) of TSP phage against clinical isolates of MRSA strains, *S. aureus*, *S. epidermidis* and other gram negative organisms.**

| **Species** | **Isolates** | **Source** | **Spot test^a^** | **EOP** |
| --- | --- | --- | --- | --- |
| ATCC 6538 strain of *S. aureus* | ATCC 6538 | ATCC | +++ | 11 |
| Methicillin Resistant *S. aureus* | MR1 | Wound swab | - | - |
|  | MR2 | CVP line | ++ | 0.005 |
|  | MR3 | Wound swab | +++ | 0.0003 |
|  | MR4 | Blood culture | - | - |
|  | MR5 | Wound swab | +++ | 5 × 10^4^ |
|  | MR6 | Pus discharge | +++ | 0.016 |
|  | MR7 | Wound swab | - | - |
|  | MR8 | Blood culture | +++ | 0.6 |
|  | MR9 | Wound swab | +++ | - |
|  | MR10 | Pus discharge | +++ | 1 |
|  | MR11 | Swab | - | - |
|  | MR12 | Wound Swab | + | - |
|  | MR13 | Blood culture | - | - |
|  | MR14 | Nasal swab | +++ | 0.065 |
|  | MR15 | Nasal swab | +++ | 0.005 |
|  | MR16 | Nasal swab | +++ | 0.005 |
|  | MR17 | Wound swab | +++ | 0.26 |
|  | MR18 | Pus discharge | +++ | 0.002 |
|  | MR19 | Diabetic foot | +++ | 53 |
|  | MR20 | Wound swab | ++ | 0.007 |
|  | MR21 | Wound C/S | ++ | - |
|  | MR22 | Wound swab | - | - |
|  | MR23 | Neck wound swab | +++ | - |
|  | MR24 | Blood | +++ | - |
|  | MR25 | Blood | +++ | 0.32 |
|  | MR26 | Pus discharge | +++ | 32 |
|  | MR27 | Pus discharge | + | 0.002 |
|  | MR28 | Pus discharge | ++ | 0.004 |
|  | MR29 | Ear swab | +++ | 0.5 |
|  | MR30 | Pus discharge | - | - |
|  | MR31 | Anterior nares | +++ | - |
|  | MR32 | Abscess | + | - |
| Methicillin Sensitive *S. aureus* | MSSA1 | Nasal swab | ++ | - |
|  | MSSA2 | Nasal swab | + | - |
|  | MSSA5 | Wound swab | + | - |
|  | MSSA6 | Catheter tip | + | - |
|  | MSSA7 | Abscess | ++ | 0.08 |
|  | MSSA8 | Nasal swab | + | - |
|  | MSSA9 | Urine | + | - |
|  | MSSA10 | Wound swab | +++ | - |
| *Staphylococcus epidermidis* | SE1 | Urine | - | - |
|  | SE2 | Eye discharge | - | - |
|  | SE3 | Urine | - | - |
|  | SE4 | Blood | - | - |
|  | SE5 | Blood | - | - |
| Gram Negative organisms | *E. coli* | UTI | - | - |
|  | *Klebsiella pneumoniae* | UTI | - | - |
|  | *Serratia marsecence* | UTI | - | - |
|  | *Pseudomonas aeruginosa* | Urine | - | - |
|  | *Acinetobacter baumannii* | RTI | - | - |
|  | *Enterobacter cloacae* | RTI | - | - |

**^a^**Spot test results (-) No lytic activity, (+) Small lytic activity, (++) moderate lytic activity, (+++) Strong lytic activity

**Table S3. Open reading frame (ORF) analysis of TSP phage**

| **ORFs** | **CDS position** | **Strand** | **Length**  **(aa)** | **Start codon** | **Function** | **Best match BLASTp** | **Identities** | **Accession No.** | **Molecular weight**  **(KDa)** |
| --- | --- | --- | --- | --- | --- | --- | --- | --- | --- |
| ORF1 | 278...679 | + | 133 | ATG | Hypothetical protein | *Staphylococcus* phage S13 | 119/133  (89%) | [BAL42329.1](https://www.ncbi.nlm.nih.gov/protein/BAL42329.1?report=genbank&log$=protalign&blast_rank=1&RID=VBXGJW9301R) | 15.2 |
| ORF2 | 693…875 | + | 60 | ATG | Hypothetical protein44AHJD_20 | *Staphylococcus* virus 44AHJD | 60/60  (100%) | NP_817315.1 | 6.9 |
| ORF3 | 882…2093 | + | 403 | ATG | Capsid and scaffold protein | *Staphylococcus* phage vB_SauP-436A1 | 402/403  (99%) | [QEA03132.1](https://www.ncbi.nlm.nih.gov/protein/QEA03132.1?report=genbank&log$=protalign&blast_rank=1&RID=VBZ1R0JF013) | 46.1 |
| ORF4 | 2108…3091 | + | 327 | ATG | Phage collar protein | *Staphylococcus* phage SCH1 | 327/327  (100%) | YP_009787916.1 | 37.9 |
| ORF5 | 3084…3839 | + | 258 | ATG | Phage lower collar protein | *Staphylococcus* phage Pabna | 256/258  (99%) | YP_009816552.1 | 29.1 |
| ORF6 | 3852…5789 | + | 645 | ATG | Putative minor structure protein  Putative major teichoic acid biosynthesis protein C | *Staphylococcus* phage S13  *Staphylococcus* phage SLPW | 626/645  (97%)  620/645  (96%) | [BAL42324.1](https://www.ncbi.nlm.nih.gov/protein/BAL42324.1?report=genbank&log$=protalign&blast_rank=1&RID=VC1CSHJD013)  YP_009278568.1 | 74.5 |
| ORF7 | 5796…6548 | + | 250 | ATG | Putative endolysin | *Staphylococcus* phage S13' | 244/253  (96%) | [BAL42323.1](https://www.ncbi.nlm.nih.gov/protein/BAL42323.1?report=genbank&log$=protalign&blast_rank=1&RID=VC48D8CZ013) | 28.6 |
| ORF8 | 6611…8056 | + | 481 | ATG | Phage tail fibers protein | *Staphylococcus* phage SCH1 | 464/481  (96%) | YP_009787912.1 | 54.7 |
| ORF9 | 8101…9876 | **+** | 591 | ATG | Major tail protein | *Staphylococcus* phage Pabna | 589/591  (99%) | YP_009816548.1 | 60 |
| ORF10 | 9878…10300 | + | 140 | ATG | Putative holin | *Staphylococcus*phage GRCS | 140/140  (100%) | [YP_009004302.1](https://www.ncbi.nlm.nih.gov/protein/YP_009004302.1?report=genbank&log$=prottop&blast_rank=1&RID=69MRNYFS01R) | 16.2 |
| ORF11 | 10275…11714 | **+** | 479 | ATG | CHAP domain-containing protein | *Staphylococcus* phage Pabna | 476/479  (99%) | YP_009816546.1 | 52.1 |
| ORF12 | 11828…14113 | **-** | 761 | ATG | DNA polymerase | *Staphylococcus* phage SCH1 | 753/761  (99%) | YP_009787907.1 | 90.2 |
| ORF13 | 14129…15376 | **-** | 415 | ATG | DNA packaging protein | *Staphylococcus* phage SCH1 | 414/415  (99%) | YP_009787906.1 | 50.3 |
| ORF14 | 15424…15906 | **-** | 160 | ATG | Hypothetical protein | *Staphylococcus* phage SCH1 | 160/160  (100%) | YP_009787905.1 | 19.4 |
| ORF15 | 16072…16485 | **-** | 137 | ATG | Hypothetical protein | *Staphylococcus* phage SCH1 | 110/137  (80%) | YP_009787904.1 | 16.4 |
| ORF16 | 16488…16667 | **-** | 59 | ATG | Hypothetical protein | *Staphylococcus* phage SLPW | 59/59  (100%) | YP_009278557.1 | 7.2 |
| ORF17 | 16718…17086 | **-** | 122 | ATG | Single-stranded DNA binding protein | *Staphylococcus* phage Pabna | 121/122  (99%) | YP_009816539.1 | 14.3 |
| ORF18 | 17110..17346 | **-** | 78 | ATG | Hypothetical protein   phiAGO13_02 | *Staphylococcus* phage vB_SauP_phiAGO1.3 | 75/78  (96%) | YP_009797772.1 | 9.2 |
| ORF19 | 17364..17666 | - | 100 | ATG | Hypothetical protein | *Staphylococcus* phage SCH1 | 95/100  (95%) | YP_009787900.1 | 11.6 |
| ORF20 | 17659..17841 | - | 60 | ATG | Hypothetical protein | *Staphylococcus* phage S24-1 | 40/60  (67%) | YP_004957416.1 | 6.9 |

**aa =** amino acids

**Table S4:** Secondary Structure composition of endolysin of TSP, SPLWand vB_SauP-436A phages

| **Secondary structure** | **TSP** | **SLPW** | vB_SauP-436A |
| --- | --- | --- | --- |
| **Helix (%)** | 15 | 11 | 13 |
| **Sheet (%)** | 35 | 34 | 36 |
| **Other (%)** | 50 | 55 | 51 |

1.
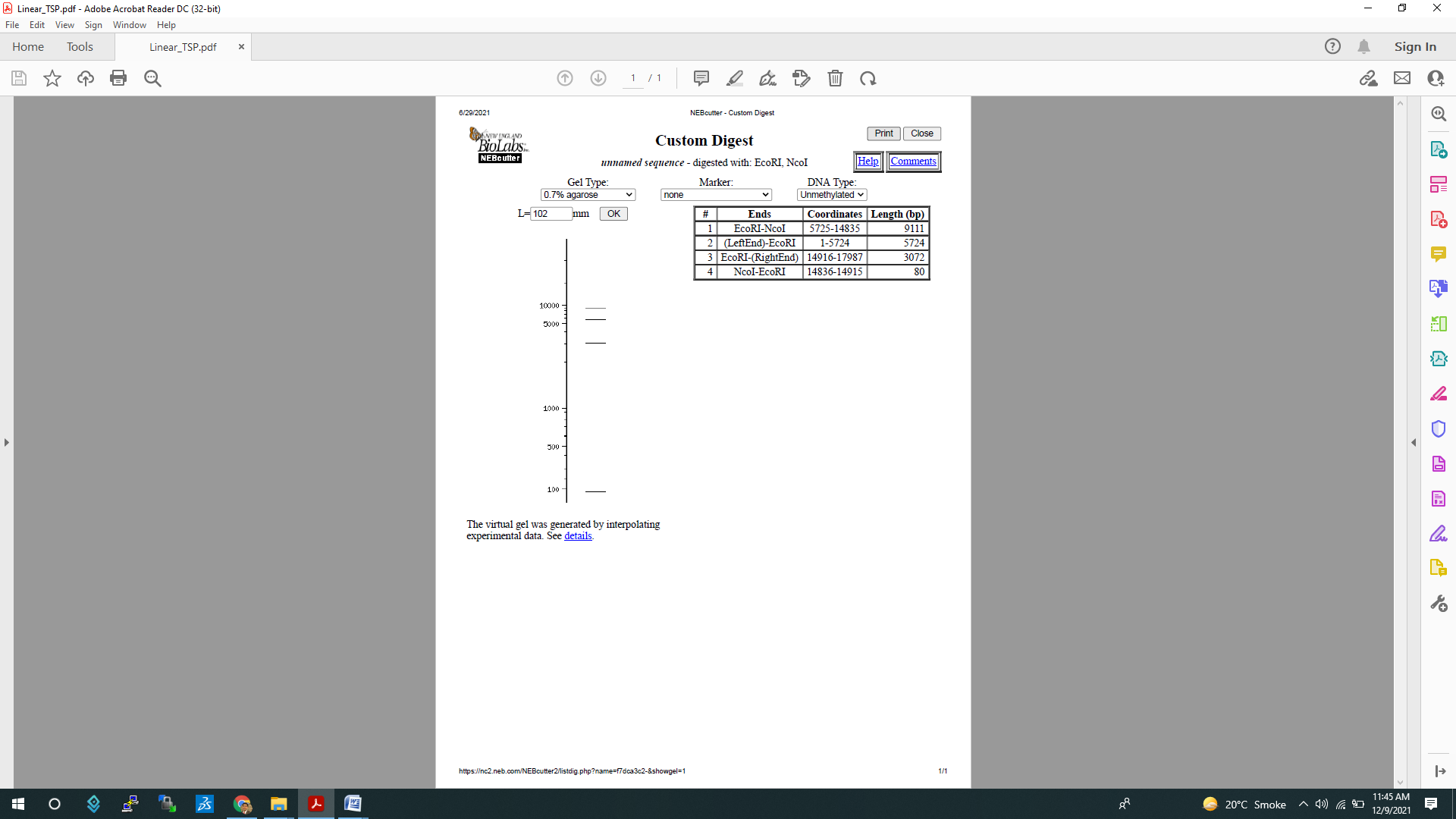

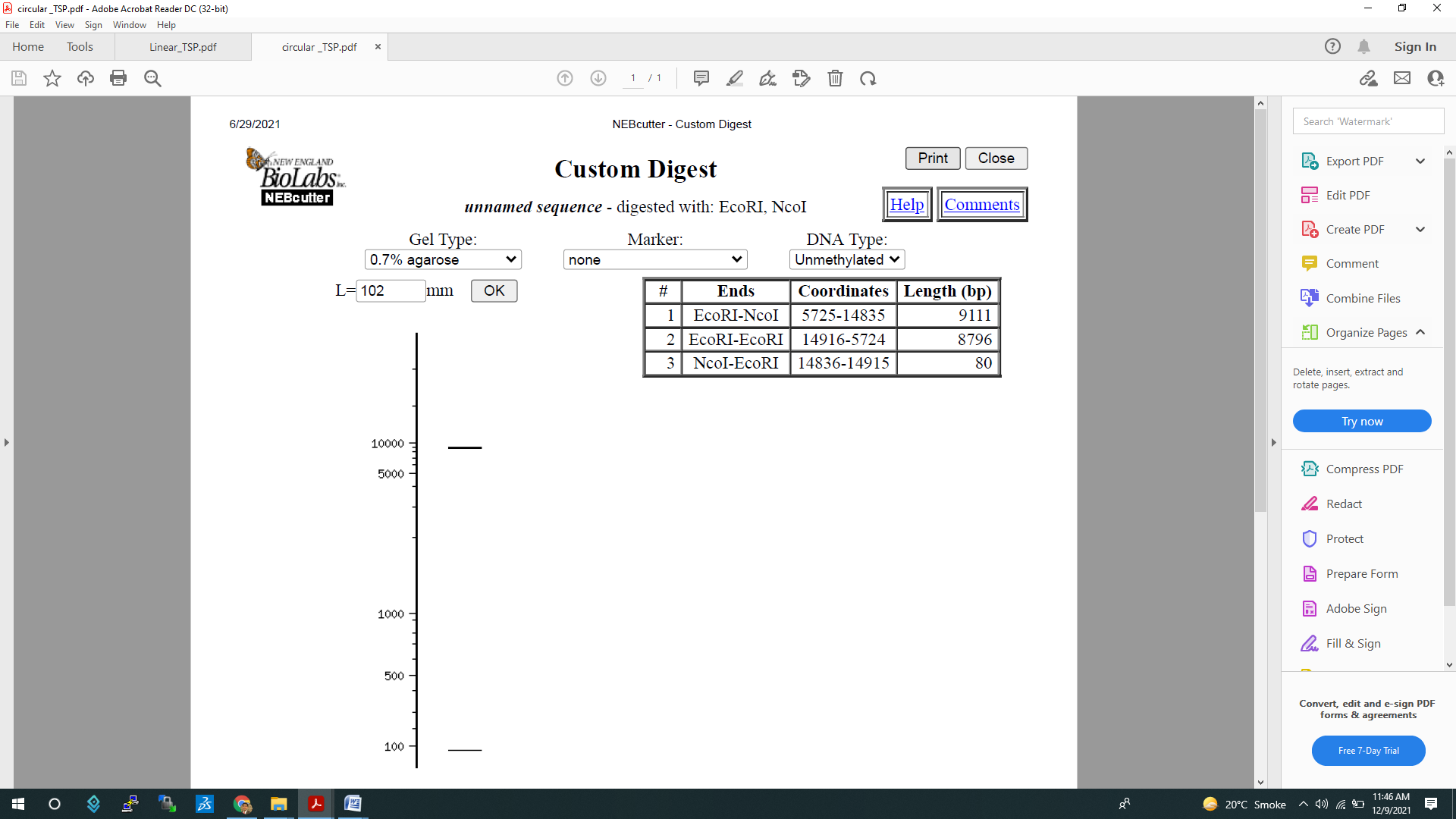
 **(B)**

**Figure S1**: Theoretical digestion pattern of TSP genome for **(B)** linear and **(C)** circular genome, determined through Neb cutter when custom digested through NcoI and EcoRI restriction enzymes.


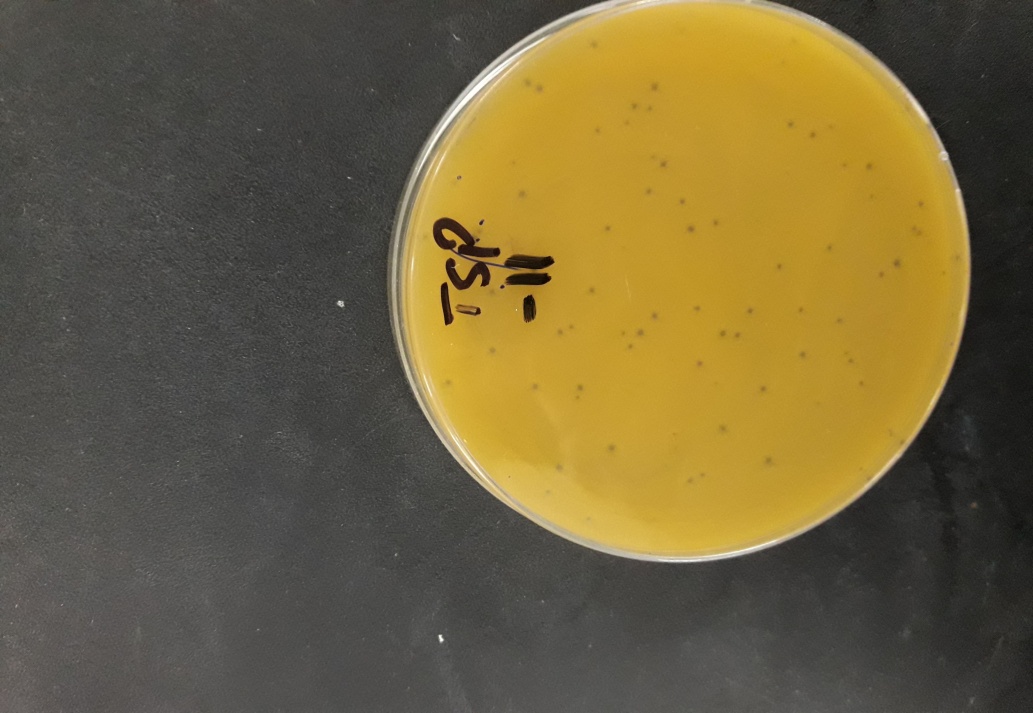

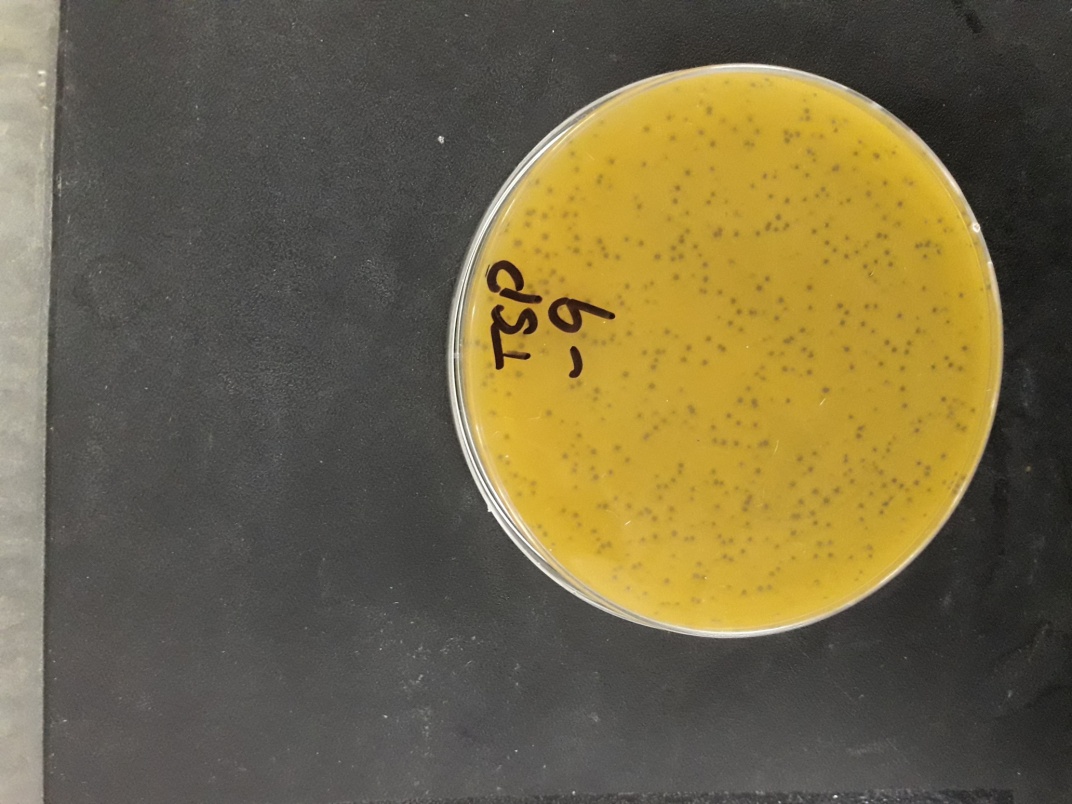

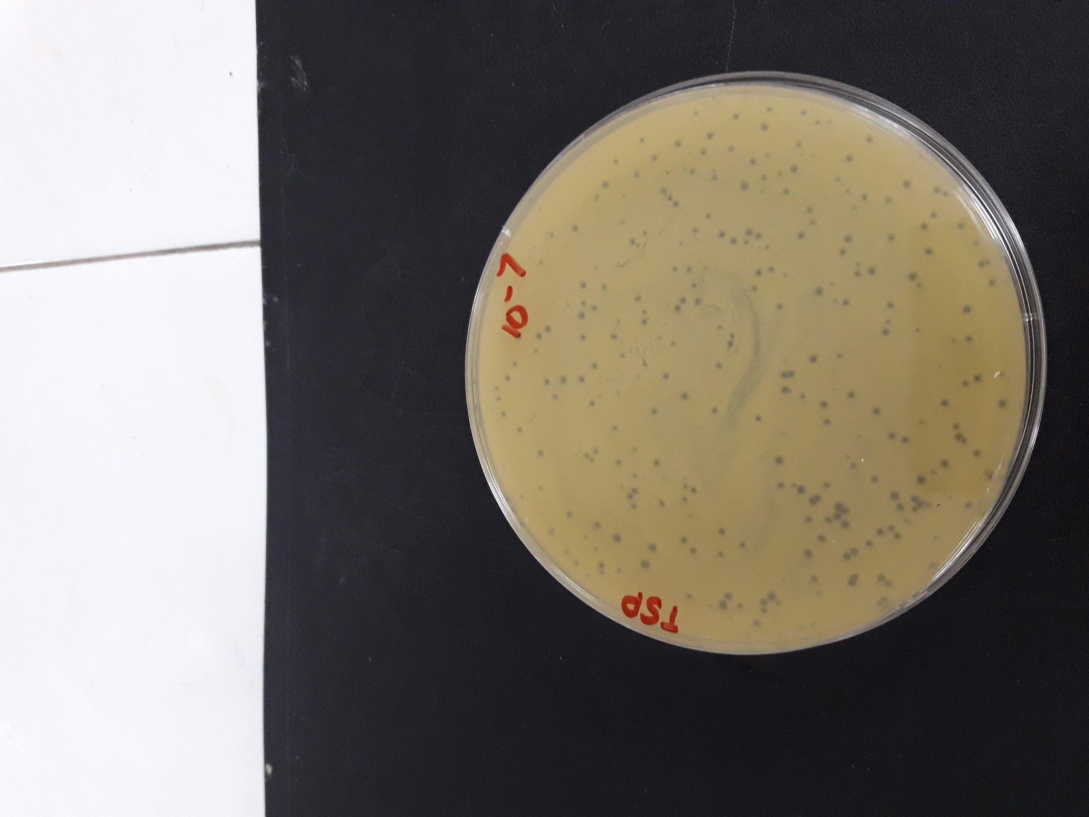


**Figure S2:**TSP phage exhibiting clear, circular and tiny lytic plaques at different dilutions i.e 10^-7^, 10^-9^ and 10^-11^ as labeled on plates.

1. (B)


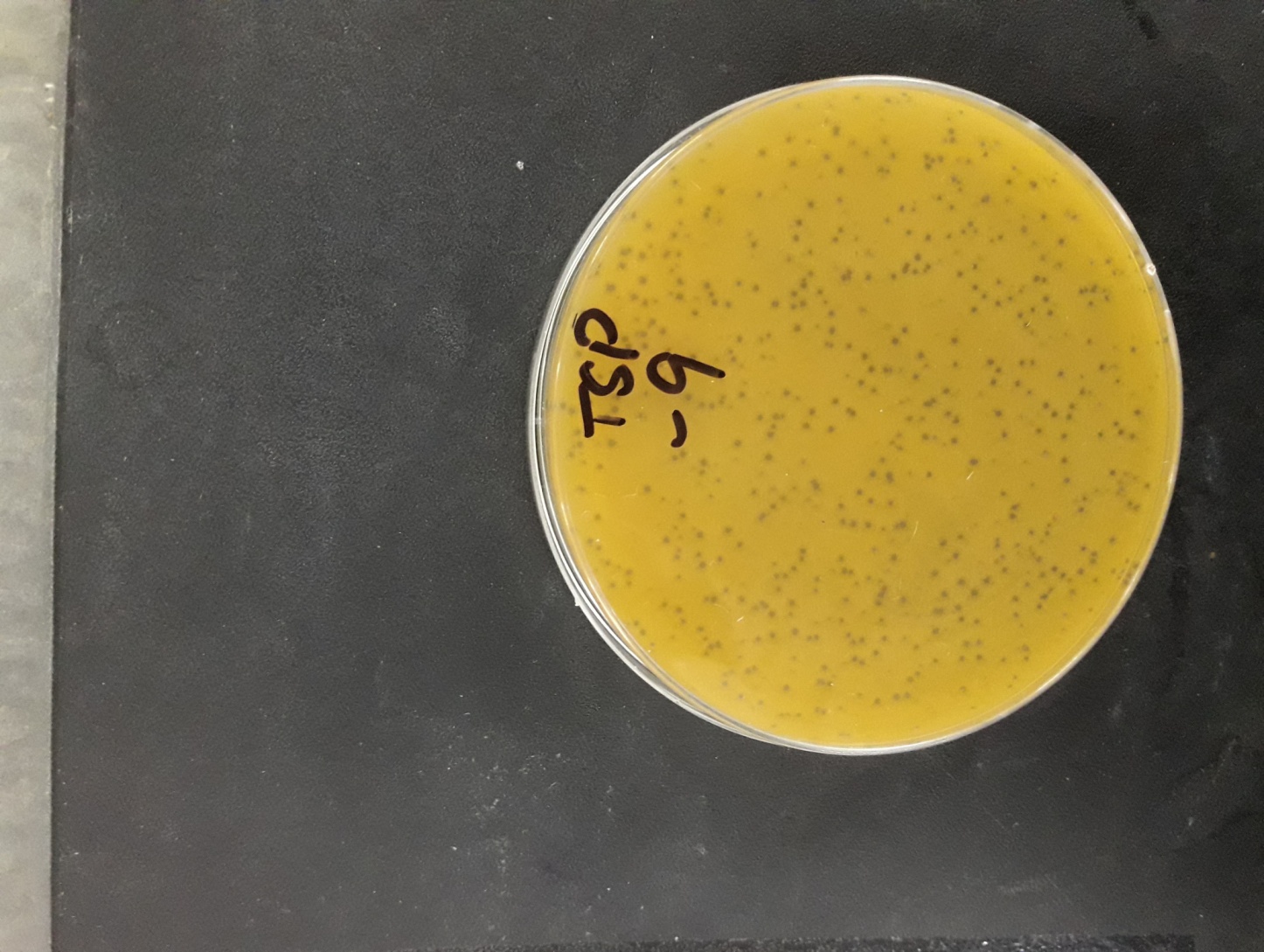

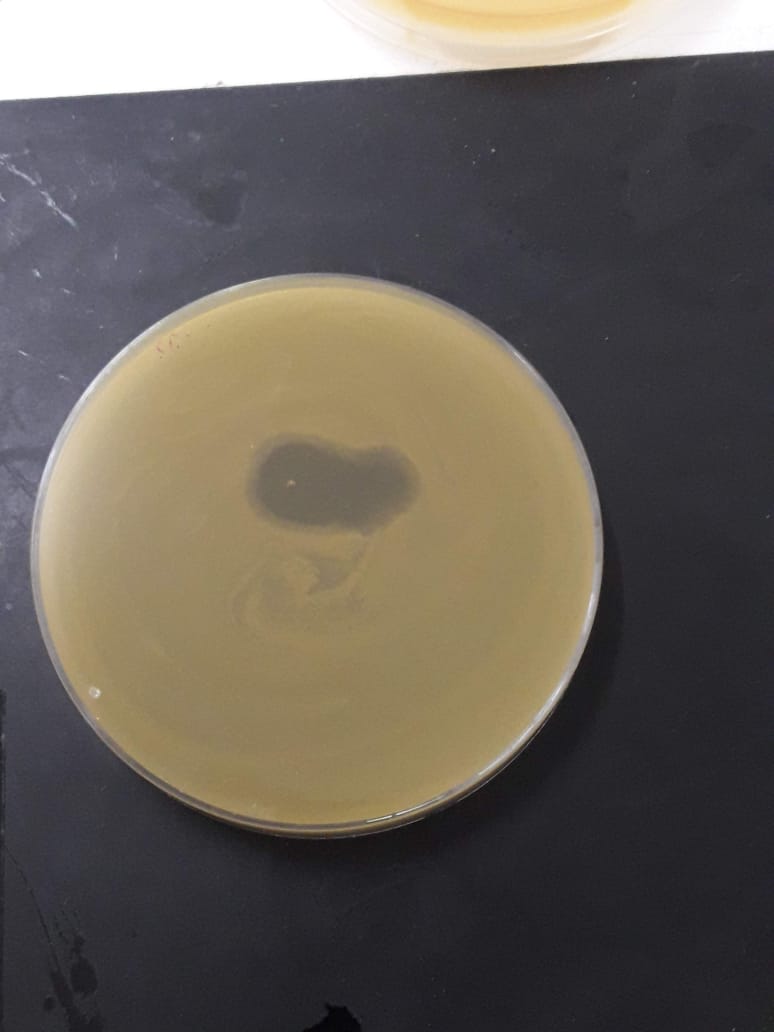


Figure S3: The uncroped agar plates showing (A) spot assay and (B) plaque assay of TSP against MR10.


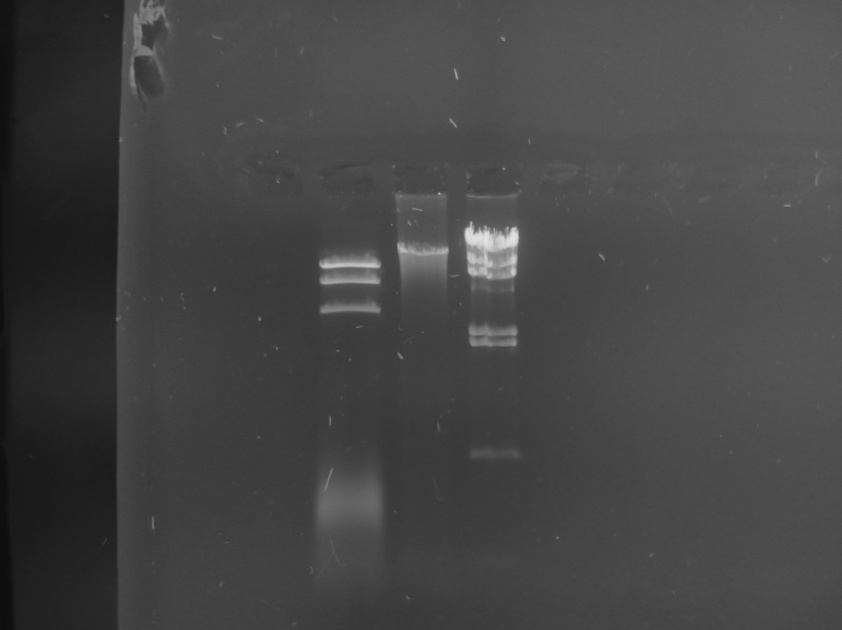


Figure S4: The uncroped agarose gel showing TSP genome digestion with NdeI and EcoRI.
